# Annotations

**Published:** 1892-10-22

**Authors:** 


### Annotations.

**A Home of Peace for the Dying.**

Seven years ago Miss Davidson opened "Freidenheim," a "Home of Peace" for the dying. She opened it with ten beds, and has maintained it at her own cost, and managed it herself until now. Can any idea appeal more tenderly to every man and woman's sympathy than this of a quiet resting place for those who are on the verge of the shadowy world, and who have no home in which to lay down the last remains of life? Many who see a husband or a wife in the last stage of hopeless illness think at once of a hospital, and suppose that for such a case the doors of every hospital in London must needs stand open night and day. But it is not so. Against the man whose days are manifestly numbered the doors, even of the hospital, are fast closed—and necessarily so. The hospital is for those who can be cured and restored to health and the power of work. It is almost incredible, but it is pathetically true, that until Miss Davidson's home was opened, just seven years ago, there was in all this vast London no single home for the dying. As showing how stern was the need for such a home, it is sufficient to state the heartbreaking fact that for every single dying person Miss Davidson has received and succoured during the last seven years she has been obliged to say "No" to at least nine other helpless applicants. For every one received, nine have had to be turned away. We sincerely rejoice, and every sympathetic reader will rejoice with us, that an effort has been successfully made to open and permanently maintain, under Miss Davidson's auspices, a much larger number of beds for the helpless dying than the original ten. It is hoped, indeed, that now that the public are invited to share in this truly necessary and honourable work, as many beds may shortly be opened as the necessities of the metropolis demand. The new "Freidenheim" will be opened by the Duchess of Teck, accompanied by the Princess Victoria, at Upper Avenue Road, N.W., on Monday, November 7th, at half-past three o'clock. The opening ceremony is to be attended by invitation. But there is no doubt that any person sympathising with the objects of the Home will be welcomed. In the meantime, we desire to commend to our readers, with deep sympathy and earnestness, this much-needed work of true charity. All communications may be addressed to Miss Davidson, Sunnyside, Upper Avenue Road, N.W.

**Drunk or Dying?**

Our special Commissioner, who recently visited the County Hospital at Exeter, was surprised and pleased to find that the question of "drunk or dying," which so frequently heads an unhappy paragraph in the lay press, had been settled in that city in the most obvious and practical way. At the Exeter Infirmary there is a separate "Emergency Ward." Any person who may be taken to the hospital in a doubtful condition, and of whom it is impossible to say whether he is "drunk or dying," is at once put into that ward; and there he is watched and tended until time settles the question of his actual condition. Some of the daily newspapers have, we are glad to see, taken up the matter, and urged upon the authorities of the London hospitals the provision of similar wards. We would like to do the same, only we feel that every argument we could use must be at least as familiar to the managers of hospitals as it is to ourselves. Nevertheless, we feel still more strongly that the honour of the hospitals, and not only of the hospitals but of medical practitioners, is deeply concerned. Our youthful house surgeons and house physicians are as a class highly meritorious. They not only possess a surprising amount of acquired knowledge; but they are usually men of very high character and excellent feeling. But even among the best class of men, a black sheep or a fool will occasionally be found; and it is a pity that such a person should have the opportunity of damaging the reputation of a whole class of honourable and worthy young men. For the sake of the public, of the honour of the hospitals, of the resident medical officers, and of the whole medical profession, we venture to urge upon all hospital authorities in London and other large centres of population, the consideration of the provision of a small "Emergency Ward" for doubtful cases.

**Experiments on Human Subjects.**

There is a distinct tendency among the younger physiologists to experiment with human subjects after experimenting upon animals wherever this is practicable. Miss Cobbe and Bishop Berry need not be alarmed. The youthful doctors have not yet taken to the practice of vivisecting criminals, or indeed of "cutting-up" anybody at all, unless it be themselves, or one another. The late Sir Robert Christison used to experiment very commonly upon himself; and it is well-known that a jug of shaving water used as an emetic once saved him from imminent death by the powerful poison of Calabar Bean. Our young experimenters are following Christison's example, and using themselves as subjects. Sometimes, also a man or two who have confidence in the soundness of their own health and in the scientific capacity of the doctors, will submit themselves to experiment "for a consideration." Dr. John Haldane and Mr. Lorrain Smith have recently carried out some experiments on these lines at the Physiological Laboratory of the University of Oxford. Their object was to determine what it is which makes the air of crowded places poisonous to those who breathe it. Is it the diminution of oxygen, as believed by some? Is it the presence of organic matter in the carbonic acid expelled from the lungs, as contended for by the majority of physiologists? Or is it the excess of carbonic acid gas pure and simple? These are practical questions of the utmost importance. Space forbids us giving so much as an outline of the experiments carried out. But they were varied, numerous, and convincing; and they were for the most part carried out on voluntary human subjects, including the experimenters themselves. The conclusions arrived at by the experimenters are several; but we wish to call immediate attention to two: the first is that it is excess of carbonic acid gas, and not want of oxygen, or the presence of organic matter in the air which causes the headache, feeling of suffocation, nausea, and other immediately alarming effects of breathing an atmosphere, contaminated by the presence of too many living persons. The second is that ventilation is not of itself sufficient to purify rooms which are constantly occupied by too large a number of individuals. To keep dwelling-rooms healthy, fresh air must be abundant and ventilation free; but in addition to this the walls and ceiling, as well as the floors, must be frequently washed with anti-septic solutions; or in cases where they are covered with paper, the paper must be much more frequently changed than it usually is, according to present custom. These it may be said are but the conclusion of sound common sense. Most true! But when the common sense is confirmed by adequate scientific experiment, even the truth is seen to be doubly true, as it certainly is doubly authoritative.

